# Artificial intelligence (AI) for virtual reality exposure therapy (VRET): A systematic review

**DOI:** 10.1038/s41398-026-03936-4

**Published:** 2026-03-26

**Authors:** Kamilla Bergsnev, Ana Luisa Sánchez Laws

**Affiliations:** 1 Psychology Institute, Faculty of Health Sciences, UiT The Arctic University of Norway, Tromsø, Norway; 2 Faculty of Education, Humanities and Social Sciences, UiT The Arctic University of Norway and Sense-IT Laboratory at the Norwegian University of Science and Technology, Trondheim, Norway

**Keywords:** Psychiatric disorders, Human behaviour

## Abstract

**Objective:**

This systematic review maps what is known about using artificial intelligence (AI) to tailor virtual reality exposure therapy (VRET) to better meet the needs of patients and therapists.

**Background:**

Exposure therapy is a well-supported treatment for fear- and anxiety-related disorders that works by exposing patients to feared or avoided stimuli. VRET can facilitate exposure that would otherwise be impractical. AI offers growing possibilities to personalize VRET, potentially improving its effectiveness.

**Inclusion criteria:**

We included peer-reviewed journal articles published up to November 14, 2025. After screening 377 records, 23 articles were included for full review.

**Methods:**

The review followed the Preferred Reporting Items for Systematic Reviews and Meta-Analyses (PRISMA) guidelines. Databases searched were PsycINFO, Web of Science, Google Scholar, EMBASE, CINAHL, and MEDLINE.

**Results:**

Studies point to promising AI applications for VRET, including conversational AI, machine learning for outcome prediction, and methods to personalize cues and contexts. However, over half of the reviewed papers in machine learning (ML) set goals or evaluated results without therapist or patient involvement.

**Conclusion:**

AI for VRET remains at an early stage. There are robust examples of best practices that integrate stakeholder perspectives, but future work should more consistently include therapists and patients early in design, development, and evaluation and should more closely integrate up-to-date theorizations on exposure/extinction. We hope this review encourages transdisciplinary collaboration in this rapidly evolving field.

## Introduction

Anxiety disorders are highly prevalent and disabling, with an estimated 359 million people affected worldwide in 2021 (approximately 4.4% of the global population) [1]. Fear- and anxiety-related disorders such as post-traumatic stress disorder (PTSD) are estimated to affect approximately 3.9% of the global population, while obsessive–compulsive disorder (OCD) has a prevalence of 1–3% [2]. Despite the availability of effective treatments, most people lack access to proper care: only about 27.6% (roughly one in four) of those who need treatment receive it [1].

Exposure therapies (ET) have strong empirical support and are central to evidence-based care for fear- and anxiety-related disorders. According to the Society of Clinical Psychology (Division 12 of the American Psychological Association), exposure-based interventions with strong support include *exposure therapy for specific phobias*, *prolonged exposure therapy for PTSD*, and *exposure and response prevention for OCD* [3–6]. Exposure is also a core component of cognitive behavioral therapy (CBT) protocols for several fear- and anxiety-related disorders, including *CBT* for panic disorder, specific phobias, and social anxiety disorder [7–11]. ET is grounded in classical conditioning and extinction learning [12]. In classical conditioning, a neutral conditioned stimulus (CS) becomes associated with an unconditioned stimulus (US). For example, a metronome sound may become associated with food after repeated pairing and, eventually, the CS alone elicits a conditioned response (e.g., salivation). When the CS is presented repeatedly without the US, the conditioned response weakens in a process known as extinction. In ET, patients are systematically and safely exposed to fear-relevant stimuli under controlled conditions to reduce maladaptive fear/anxiety responses and their consequences [13, 14].

Despite strong evidence for ET, only an estimated 10–30% of therapies in clinical practice employ exposure [15], with similarly low use reported in youth anxiety treatment [16]. Barriers include patient and therapist concerns about potential harm of direct exposure to the feared stimuli, as well as practical challenges related to cost, time, and logistics [15, 17, 18]. For example, it could be difficult for a therapist administering an in vivo exposure for fear of flying to join the patient in an actual plane flight, because of scheduling problems or because of cost.

Virtual reality exposure therapy (VRET) is one way to address these barriers. VRET adds a virtual reality component to the therapist’s toolkit. Virtual reality (VR) uses computer-generated content (audio, video, motion graphics, 3D objects) delivered through audiovisual and haptic technologies (e.g., head-mounted displays or immersive room projection systems) to create a compelling sense of presence in a simulated environment. VR affords high flexibility in designing exposure scenarios. Three-dimensional reconstructions of physical space allow users to navigate and interact with the environment in real time [19], enabling patients to virtually experience feared situations (e.g., approaching a spider, looking down from a tall building, speaking in public). While VR cannot fully replace real-world exposure, it can reproduce key features of in vivo settings. Other computer-based approaches (e.g., augmented reality exposure therapy, ARET) exist, but this review focuses on VRET because of qualitative differences in the user experience between the AR and VR. AR overlays digital elements onto the user’s physical environment, whereas VR fully immerses the user in a separate, simulated space.

Since early case studies in the 1990s [20], evidence has accumulated showing that VRET achieves outcomes comparable to other exposure-based treatments across several phobias and anxiety-related conditions, with practical advantages when in vivo exposure is impractical. Recent reviews and meta-analyses [21–40] generally support VRET’s efficacy and effectiveness and identify research needs across age groups and diagnostic subtypes (see overview in Table S1 in the supplementary materials). Based on current evidence, the American Psychological Association recognizes VRET as an acceptable modality of exposure therapy when in vivo exposure is not feasible (e.g., fear of flying).

A persistent clinical gap concerns personalization and scalability. Even when VRET is available and effective, tailoring exposure intensity and context in real time remains labor-intensive and highly dependent on therapist expertise. Therapists must define appropriate schedules or hierarchies for exposure, monitor safety and engagement, and adapt stimuli to individual cues (e.g., avoidance, dissociation, physiological arousal). These demands create scalability and equity challenges, potentially limiting access to optimally administered exposure outside specialized centers or when sessions are delivered remotely. In many current VRET systems, therapists supervise simulations in real time and manually adjust stimuli or environmental features based on mirrored displays, sensor data, and patient feedback. Prior work has leveraged physiological signals to support non-AI automation—patient-driven control via biofeedback, dynamic environment modification using conventional computational methods, and therapist-driven control guided by real-time physiological monitoring [41–48]—but systematic, patient-specific personalization and therapist decision support remain incompletely addressed.

Recent advances in artificial intelligence (AI) offer potential solutions to many of the personalization, equity and scalability challenges mentioned above. When it comes to personalization, AI is now being tested to provide real-time biofeedback to patients and to therapists supervising VRET, to serve as a screening tool to determine appropriate exposure levels, and to predict posttreatment benefit. In terms of equity and scalability, Conversational AI agents using generative AI are being explored to provide realistic, intuitive interactions within VR environments, and knowledge-based/hybrid AI approaches are being investigated to improve the adaptability, transparency and simulation control of AI used in VRET systems (see the Technical AI Terminology provided in the methods section).

However, despite the promises of these technologies in solving the aforementioned challenges, the integration of AI into mental health care must be done responsibly. Critical aspects to consider include establishing the absolute limits of AI (when is it unsafe to use AI?), identifying which patient populations and clinical presentations that respond well to AI-driven support, and which do not, assessing the quality of care provided by AI with and without human supervision, and assessing how AI tools meet the needs of underserved populations without further entrenching inequities.

Accordingly, the objective of this systematic review was to evaluate and synthesize the existing evidence on how AI is being used to tailor and deliver VRET to better meet the needs of patients and therapists. Our research questions were: (1) Which patient- and therapist-facing needs are currently addressed by AI-enabled VRET? (2) What clinical applications, benefits, and challenges are reported? We also examined whether applied studies integrate contemporary exposure/extinction theory and stakeholder input.

We describe our PRISMA-based methods, then present results in three clusters—machine learning, conversational AI, and knowledge-based/hybrid AI—using parallel subheadings (study aims and AI role; theory use; stakeholder involvement; main results and added value; study limitations; implications for clinical use), followed by discussion and recommendations.

## Method

### Technical AI terminology

In this review, we use AI as an umbrella term spanning knowledge-based systems (e.g., formally encoded knowledge bases), machine learning (ML; automated pattern discovery from data), representation learning (automatic feature discovery), and deep learning (composing complex concepts from simpler representations) [49]. ‘Conversational AI’ (also called chatbots) refers to a software system that interacts with users through natural language—via text and/or speech—to understand inputs, manage a dialogue, and generate context-appropriate responses. ‘Generative AI’ (also known as GenAI) refers to models that learn the statistical structure of data (the data distribution) and can produce new, synthetic content that resembles the training data. Unlike discriminative models that classify or predict labels, generative models synthesize outputs—text, images, audio, video, code, or 3D objects—often conditioned on a prompt or other inputs. Knowledge-based AI refers to systems that represent domain knowledge explicitly (e.g., rules, ontologies, knowledge graphs) and use symbolic reasoning to draw conclusions or recommend actions. Hybrid AI combines knowledge-based methods with data-driven learning (e.g., machine learning) so that learned models handle perception/estimation and/or tune parameters, while explicit rules and structures govern decision-making, constraints, and explanations. A short literature list on AI basic concepts, definitions and techniques is provided in the Supplementary Materials.

### Study design

The reporting of this systematic review was guided by the standards of the Preferred Reporting Items for Systematic Review and Meta-Analysis (PRISMA) Statement [50, 51].

### Eligibility, inclusion and exclusion criteria

We selected studies investigating AI for VRET. The search included the following databases: PsycINFO (OVID), Web of Science, Google Scholar, Medline (OVID), CINAHL and EMBASE. Both authors were involved in reviewing the articles and deciding which ones to keep.

#### Inclusion criteria

To be included in the review, papers needed to focus on VRET or in the use of VR within an exposure/extinction paradigm for the treatment of fear- and anxiety-related disorders, as described in the above literature review. Peer-reviewed journal papers were included if they were: published up until November 14, 2025, involved human participants, and included the development of AI for VRET. Quantitative, qualitative and mixed-method studies were included. Only peer reviewed journal articles were included. No language filters were used.

#### Exclusion criteria

Articles that dealt with pharmacology, non-peer reviewed publications, conference papers and dissertations, protocols, review articles, preprint articles and animal studies were excluded. Papers were also excluded if they did not use AI or did not apply it to a VRET-like setting.

### Search strategy

The search strategy and terms used were *virtual reality exposure therapy*, *VRET, automation, machine learning, artificial intelligence, customization, personalization*, and *biomarkers* respectively. The steps in the controlled search are presented in Table 1.

**Table 1:** Search strategy.

Database	Search strings
**PsycINFO (OVID)**	APA PsycInfo <1806 to November 2025 Week 2> 1 (adult* or civilian* or veteran* or patient* or human* or person* or participant*).mp. 3582346 2 exp virtual reality exposure therapy/ or VRET.mp. 437 3 exp automation/ or exp biofeedback/ or exp machine learning/ or exp artificial intelligence/ or exp customization/ or exp personalization/ or exp biomarkers/ 138282 4 1 and 2 and 3 15 5 4 and “Peer Reviewed Journal”.sa_pubt. 13Results: 13
**Web of Science**	(ALL = (adult) OR ALL = (human) OR ALL = (participant) OR ALL = (civilian) OR ALL = (patient) OR ALL = (veteran) OR ALL = (person)) AND (ALL = (virtual reality exposure therapy) OR ALL = (VRET)) AND (ALL = (automation) OR ALL = (biofeedback) OR ALL = (machine learning) OR ALL = (customization) OR ALL = (personalization) OR ALL = (biomarkers) OR ALL = (artificial intelligence)) Results: 171 Filtered by Document Type “Article”: 97 Results: 97
**Google Scholar**	(machine learning or artificial intelligence or deep learning) AND (virtual reality exposure therapy or vret) and (peer reviewed) -(systematic review) -(narrative review) -(meta analysis) Results: 224
**Medline (OVID)**	Ovid MEDLINE(R) and Epub Ahead of Print, In-Process, In-Data-Review & Other Non-Indexed Citations, Daily and Versions <1946 to November 14, 2025> 1 (adult* or civilian* or veteran* or patient* or human* or person* or participant*).mp. 26190633 2 exp virtual reality exposure therapy/ or VRET.mp. 1339 3 exp automation/ or exp biofeedback/ or exp machine learning/ or exp artificial intelligence/ or exp customization/ or exp personalization/ or exp biomarkers/ 1274702 4 1 and 2 and 3 62 5 4 and “Journal Article”.sa_pubt. 59 Results: 59
**CINAHL**	(adults OR human OR participants OR civilian OR patient OR veterans OR person) AND (virtual reality exposure therapy OR vret) AND (automation OR biofeedback OR machine learning OR artificial intelligence OR customization OR personalization OR biomarkers) Results: 34
**EMBASE**	Embase Classic+Embase <1947 to 2025 November 13> 1 ((adults or human or participants or civilian or patient or veterans or person) and (virtual reality exposure therapy or vret) and (automation or biofeedback or machine learning or artificial intelligence or customization or personalization or biomarkers)).mp. [mp=title, abstract, heading word, drug trade name, original title, device manufacturer, drug manufacturer, device trade name, keyword heading word, floating subheading word, candidate term word] 77 2 1 and “Article”.sa_pubt. 37 Results: 37

### Data extraction and synthesis strategy

Data from the included studies (n = 23) were extracted and coded into a matrix. Data extraction was performed in Google Sheets. The researchers jointly prepared a spreadsheet based on PRISMA items. Each study was assigned a unique identifier for coding purposes. After full-text screening, it was evident that the studies were heterogeneous, with substantial differences in participant characteristics, goals, AI approaches, and methodological quality.

Following recommendations in [52], we developed a second qualitative synthesis matrix to combine deductive and inductive thematic analyses. For deductive (theoretical) thematic analysis, we predefined themes based on (a) our prior work (VRET personalization [53]), (b) our research questions (needs related to patients and therapists and what is known about clinical applications) and (c) current theory (relation to established frameworks for extinction and exposure). In inductive thematic analysis, the process is data-driven; the goal is to identify themes that the authors of the reviewed articles themselves flagged as important [54]. This review combines both approaches. Our thematic matrix therefore included the following deductively generated themes: “Study aims and AI role,” “Theories or models of extinction/exposure used,” and “How patients’/therapists’ perspectives were included”. In addition to these top-down themes, we created an inductive set of themes based on codes derived from key topics in the reviewed articles: “Main results and added value,” “Implications for clinical practice,” and “Study limitations.” The presentation of results follows the themes of this second matrix.

### Risk of bias (RoB) assessment

Given the heterogeneous set of studies, we compiled risk-of-bias guidance from JBI to inform our assessment [55–58]. We defined High Risk of Bias (High RoB) as studies meeting 50% or fewer of the criteria; Medium Risk of Bias (Medium RoB) as studies meeting more than 50% but less than 75% of the criteria; and Low Risk of Bias (Low RoB) as studies meeting at least 75% of the criteria. Using these thresholds, we classified 9 studies as Low RoB (robust methods and reporting), 9 as Medium RoB (some lack of clarity in methodological descriptions), and 5 as High RoB (inconsistencies in reporting and/or application of methods) (see Supplementary Materials).

For reporting, we considered relevant guidance on handling High RoB studies [59–61]. We present a primary analysis restricted to Low and Medium RoB studies and a sensitivity analysis [62] that includes High RoB studies. Excluding High RoB studies from the primary analysis is intended to prevent propagation of potentially erroneous findings; including them in the sensitivity analysis allows us to assess the robustness of conclusions and provide a complete, transparent account of available research.

### Heterogeneity of studies

We opted for a broad review due to the scarcity of articles specifically addressing AI for VRET—a reasonable limitation given the cutting-edge nature of these technologies. This breadth, however, introduced substantial heterogeneity across studies and approaches.

We observed variation in clinical factors (participants, outcomes, interventions), methodological features (study design, measurement tools, risk of bias), and statistical choices (outcome evaluation), all of which could influence findings through under- or overestimation of effects [63]. Because heterogeneity precluded meta-analysis, we prespecified a clustered narrative synthesis using three categories: (a) machine learning (ML) studies, (b) conversational AI studies, and (c) knowledge-based/hybrid AI studies. This clustering helps avoid conflating findings from fundamentally different study types while providing a broader overview of AI in VRET. We used a primary analysis limited to Low/Medium RoB, and a sensitivity analysis for High RoB ML studies This also mitigates concerns about evidence quality related to High RoB studies, which occurred only in the ML cluster.

## Results

**Fig. 1:**
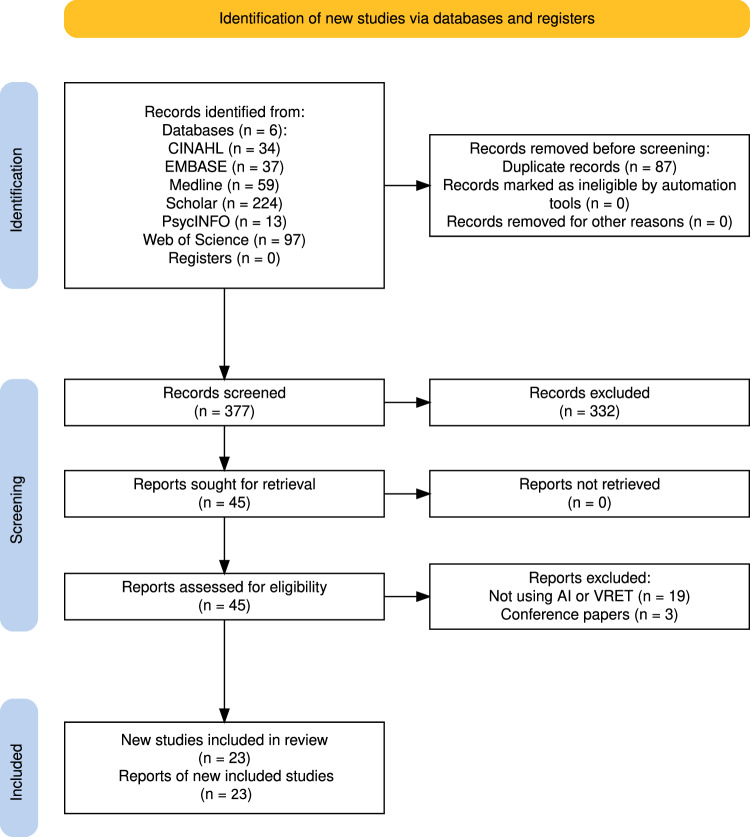
PRISMA Flow Diagram. The figure presents the results after search and screening, including the final number of studies assessed.

Figure 1 presents the results of the search as a PRISMA Flow Diagram.

The total number of records found across the six search databases was 494, of which 377 records were screened after removing duplicates. Of these, 45 records were found eligible after the first round of screening, with 23 reports selected for full review. Articles were in general cross-disciplinary work at the intersection of clinical mental health, neuro/physiological sensing, and machine learning for VR systems. Geographically, there was strong representation from East Asia and Europe, with targeted contributions from North America (notably PTSD research). Because study designs and aims varied substantially, we present results in three clusters— machine learning (ML), conversational AI, and knowledge-based/hybrid AI—using parallel subheadings and focusing on clinical added value.

### Primary analysis

Primary analysis covers: **(1) machine learning (ML) studies** [64–74], **(2) conversational AI studies** [75–78], and **(3) knowledge-based/hybrid AI studies** [79–81]. High risk of bias (High RoB) studies [82–86] occurred only in the ML cluster. A sensitivity analysis including the High RoB studies is presented at the end of the ML section.

#### Machine learning (ML) studies

Table 2 below summarizes ML studies.

**Table 2:** Machine learning (ML) studies.

Studies included in primary analysis	
Authors / year	Aims
**Low RoB**	
Jung et al. [64]	Researchers evaluated whether ML could be used to classify patients with panic disorder and agoraphobia based on multimodal data (heart rate variability HRV, skin conductance response SCR, and self-reported anxiety) collected during VR exposure.
Chavanne et al. [65]	An ensemble learning approach was used to perform a binary classification prediction between responders and non-responders at posttreatment. The purpose was to assess if machine learning applied to this type of data could aid in predicting VRET posttreatment outcomes. The authors also examined another 2nd-level classifier, soft voting with the sum of 1st-level predictions.
Cheng et al. [66]	Authors designed a voting classification algorithm, an ensemble learning-based classification algorithm that uses a majority vote to determine the final classification, to classify acrophobia and non-acrophobia movement data in a VRET environment. The aim was to use the model to classify participants into “acrophobic” and “non-acrophobic” based on movement data.
de With et al. [67]	ML models were used as subject-dependent and subject-independent classifiers. The classifiers were trained and tested with the experimental group using leave-one-subject-out cross-validation.
Leehr et al. [68]	ML models were used on data from reported follow-up VRET outcomes (after 6 months) to compare model accuracy between two study sites. The approach was cross-validation, where the data from one site were used for training and from the other site for testing, and vice versa.
**Medium RoB**	
Park et al. [69]	ML algorithms were developed to predict the upper tertile group of various anxiety symptoms based on multimodal data from VRET sessions for social anxiety disorder (SAD) patients.
Apicella et al. [70]	Data-fusion-based strategies for ML methods were investigated for real-time assessment of fear of heights intensity in VRET for acrophobia.
Apicella et al. [71]	The purpose was to investigate an electroencephalography (EEG)-based classification system of three levels (high, medium, low) of fear of heights in VRET.
Goel et al. [72]	ML was used to investigate accurate classification of internal brain stress levels using fMRI while Veterans were exposed to VRET. The goal was to automate and thereby facilitate fMRI neurofeedback-associated therapies.
Chun et al. [73]	K-means clustering, a representative detached clustering algorithm that uses unsupervised learning, was applied to label participants as belonging to the severe and non-severe symptom groups respectively. Different classification models were conducted to train the models on the data.
Handouzi et al. [74]	Authors used machine learning and Blood Volume Pulse (BVP) in short-time recognition windows (4 seconds) to classify participants in two states (anxious or not anxious) and three states (no anxiety, anxiety level 1, anxiety level 2).
**High RoB (Studies used for sensitivity analysis)**	
Mevlevioğlu et al. [82]	Train–test split was applied to compare different classification methods. The dataset was split into 80% training data and 20% testing data using the GroupShuffleSplit method.
Rahman et al. [83]	Different Machine Learning models were tested to classify publicly available datasets on stress and then use the classifier in a session with healthy participants to display biofeedback within the VRET.
Petrescu et al. [84]	Authors used EEG data from a VRET session to compute functional connectivity between each pair of channels to obtain functional brain networks (FBNs). They then trained a set of machine learning (ML) algorithms and convolutional neural networks (CNNs) with FBNs directly as inputs to classify participants as having low, mild or high anxiety.
Bălan et al. [85]	Authors investigated machine learning and deep learning sequential models for binary and multi-class classification. The models were used for two classifiers, one predicting fear level based on physiological measurements, and one predicting the VR game level to be played next.
Šalkevicius et al. [86]	Authors used machine learning and early signal fusion (where signals from all modalities are mixed and go through the feature selection stage as a combined dataset) with data from GSR, BVP and skin temperature, to classify participants within four different levels of anxiety.

##### Study aims and AI role

Most studies in the primary analysis (11 studies) employed machine learning (ML) [64–74], with applications primarily framed as classification tasks. Overall, the ML studies aimed to improve the efficiency of VRET by (i) providing biofeedback to patients, (ii) offering diagnostic or decision-support tools for therapists, and (iii) facilitating real-time therapy monitoring.

##### Theories or models of extinction/exposure used

Most ML studies [64–67, 70–72, 74] did not provide background on exposure or extinction theory. One study [65] formed part of a broader clinical program in which related papers detail the theoretical foundations. Study [68] referenced literature on neurocognitive correlates of pretreatment experimental fear extinction and associated inhibitory learning processes as predictors of response to subsequent exposure. Study [69] briefly drew on affective neuroscience to discuss measuring mental states via physiological signals. Study [73] conceptualized exposure as fear reduction.

##### How patients’/therapists’ perspectives were included

Five ML studies [64, 65, 68, 72, 73] were developed or tested in collaboration with therapists or patients. One study [64] was conducted by a researcher with a master’s degree in psychology under the supervision of a board-certified psychiatrist. Two studies [65, 68] were part of multisite clinical trials conducted by the *Collaborative Research Centre Fear, Anxiety, Anxiety Disorders* (CRC TRR 58), a translational network spanning Münster, Hamburg, Mainz, and Würzburg. One study [72] used an in vivo hierarchy procedure to co-develop VR stimuli between each veteran and the clinician before the first test session. One study [73] was conducted at a university hospital. In the remaining six studies [66, 67, 69–71, 74], aims were defined by AI researchers. One study [66] aimed to provide biofeedback to patients, and five [67, 69–71, 74] aimed to enable system personalization for automated treatment delivery.

##### Main results and added value

ML applications in VRET primarily target three use-cases: (1) outcome prediction (e.g., responder/non-responder, symptom severity), (2) state classification for exposure schedule and biofeedback (e.g., fear/anxiety levels), and (3) neurofeedback frameworks. Table 3 below summarizes key findings in each category, focusing on whether AI added value beyond clinical or chance baselines and on generalizability.

**Table 3:** Main results and clinical added value for ML studies.

Study	Condition	Data/Modality	Performance (metrics)	Clinical added value	Limitations/Notes
**Use-case 1: Outcome prediction**					
[65]	Spider phobia	Clinical questionnaires, sociodemographics, (f)MRI including BOLD-signal variance	Most modalities at chance; BOLD-signal variance above chance. Posttreatment ensemble accuracy 0.60–0.63; no modality predicted follow-up outcomes	Limited added value; cautious generalizability	Adding (f)MRI did not improve over clinical/sociodemographic data; generalizability cautioned
[68]	Mixed (primary outcome at posttreatment)	Single-case ML across sites	Low but statistically significant mean accuracy within combined sample; balanced accuracy non-significant between sites; failed to predict remission rates	Minimal added value; highlights multi-site accuracy challenges	Between-site generalization poor; remission prediction failed
[69]	Social anxiety disorder (SAD)	CatBoost on multimodal data (physiology + acoustics + session features)	AUROC 0.852 (severe social phobia) and 0.866 (rumination); outperformed single-modality inputs	Potential aid for tailoring exposure schedules	Requires external validation
**Use-case 2: State classification & biofeedback**					
[64]	Panic/agoraphobia	Various classifiers; VR-stimulus settings; medication status	Specificity/precision ≥0.80; sensitivity up to ≥0.82; performance stable across parameters, VR settings, and medication	Candidate diagnostic aid	External validation needed
[66]	Acrophobia (fear of heights)	Sparse joint multimodal data	Highest classification accuracy 94.6%	Candidate diagnostic aid	External validation needed
[67]	Not specified (subject-dependent vs. independent)	Subject-dependent vs. subject-independent models	Approx. accuracy: 70% (subject-dependent) vs. 74% (subject-independent); subject-independent generally better	Candidate diagnostic aid	External validation needed
[73]	Social anxiety disorder (SAD)	Two ML models; F1-based evaluation	Models predicted severity levels for two SAD subdomains (F1-weighted evaluation)	Candidate diagnostic aid	External validation needed
[70]	Fear of heights (VRET)	EEG + ECG	Accuracy 58%	Modality choice matters	Performance modest
[71]	Fear of heights	EEG bands (3-level fear classification)	Mean accuracy 86.10 ± 8.29%	Modality choice matters	Same group as [70]; improved with EEG band classification
[74]	Anxiety (slight to moderate; n = 20)	BVP-based short-window classification	Accuracy: 95.72% (2-state: no anxiety vs. anxious); 94.91% (3 levels: no anxiety, level 1, level 2)	Working proof-of-concept in small samples	Generalizability uncertain; small sample
**Use-case 3: Neurofeedback frameworks**					
[72]	Veterans’ stress	Multisession fMRI; ML (3 algorithms; deep learning best)	Deep learning RMSE 0.6 ± 0.1 (mean ± SE)	Supports feasibility of individualized real-time neurofeedback; promising framework	Resource-intensive; clinical gains not yet demonstrated

##### Implications for clinical practice

In study [64], the authors suggested that physiological measures could serve as feasible diagnostic aids for panic disorder and agoraphobia. In study [65], the authors proposed exploring additional psychological, neuroimaging, and biological measures—such as early response to psychotherapy and early changes in functional connectivity—for individual-level predictions, and investigating epigenetic markers with ML to identify group-level biomarkers of psychotherapy response. In study [67], authors argued that the feasibility and ecological validity of combining VR-HMDs with fNIRS to elicit and detect fear responses could pave the way for brain–computer interfaces to manipulate and control VRET.

Study [68] advised caution when using ML for pre- or posttreatment assessment, noting limited clinical utility of clinical and sociodemographic predictors for ML classification; however, they encouraged exploration of multimodal predictors. This position aligns with study [69], where an ML algorithm using integrated multimodal data predicted upper-tertile anxiety symptoms in SAD with higher performance than acoustic or physiological data alone from a VRET session, supporting clinical use of multimodal data.

In study [72], the authors proposed that their framework for preprocessing whole-brain cortical fMRI and training ML models across sessions could enable individualized, real-time fMRI neurofeedback during VRET for PTSD.

##### Study limitations

Study [65] noted that their sample (n = 190) comprised relatively homogeneous spider-phobic patients without major comorbidities, limiting generalizability to the broader anxiety population. Authors also observed that high classification accuracies reported in earlier literature often stemmed from single-site, small samples with poor generalizability, and called for more representative samples even if predictive performance declines. In study [68] (n = 172 from the same program as [65]), the authors remarked that, despite larger sample sizes than many prior studies, the dataset remained small for ML. They suggested that larger, independent multisite studies could improve reliability but noted that some studies still achieve only ~60% accuracy with substantially larger samples. Their assessment was that large-scale multicenter studies may yield more stable prediction models, but increasing sample size alone can produce qualitatively different performance metrics. They further reported that their ML models and sociodemographic predictors did not meet two prerequisites for clinical utility—high predictive performance and generalizability—suggesting that multimodal predictors may be more promising.

##### Summary of primary analysis findings

Across ML studies, outcome prediction showed modest and fragile gains over clinical baselines; multimodal inputs sometimes improved classification of severe symptom groups. State classification/biofeedback is feasible across several sensors but requires external validation and diversity to ensure equity. fMRI-based neurofeedback frameworks are promising but resource-intensive. Overall, added clinical value is clearest when multimodal data are used and when models are externally validated.

### Sensitivity analysis for ML studies

As noted earlier, the sensitivity analysis for machine learning (ML) includes five studies rated as High risk of bias (High RoB) [82–86]. These studies were small/convenience samples and lacked stakeholder involvement. Findings are best viewed as technical proofs-of-concept rather than clinically actionable evidence. Overall, aims in these High RoB ML studies mirrored those in the primary analysis: providing biofeedback, diagnostic/decision-support tools, or facilitating real-time therapy—without involving patients or therapists in defining study goals. References to emotion theories (Ekman [75], Russell [76]) were present in [84] and [85]; such references were absent from Low RoB studies. Results claiming high algorithmic performance should be interpreted with care given the low sample sizes across these studies. Table 4 summarizes the main findings for these studies, following the categories outlined for the primary analysis.

**Table 4:** Main findings for studies in the sensitivity analysis group.

Study	Study aims and AI role	Theory background	Patient/therapist involvement	Study limitations	Main results, added value, and clinical practice implications
[82]	Improve comfort and objectively measure user anxiety to increase ease of use for patients and therapists; anxiety classification model	N/A	N/A	Convenience sample, *n* = 29 (university campus) Small, convenience sample; limited generalizability	Classified participants as having “No anxiety” 80% correct; “High anxiety” 76% correct; “Mild anxiety” 69% correct, demonstrating the potential of using ML for symptom level classification
[83]	Biofeedback targeted for patients; model designed to predict arousal	N/A	N/A	Validated on public datasets: SWELL (*n* = 25); “Stress classifier with AutoML” (*n* = 17); “Electroencephalograms during Mental Arithmetic Task Performance” (*n* = 36 healthy) Validation on small public datasets	Suggested detection of imminent meltdowns in ASD and other conditions; early intervention to reduce arousal/prevent escalation
[84]	Automate adjustment of exposure level during VRET	Brief therapeutic overview of exposure; no underlying theory conceptualized. Ekman’s basic emotions [75]; Russell’s circumplex model of affect [76]	N/A	N/A	N/A
[85]	Evaluate EEG suitability for real-time classification of agoraphobia during VRET	Brief therapeutic overview of exposure; no underlying theory conceptualized. Ekman’s basic emotions [75]; Russell’s circumplex model of affect [76]	N/A	*n* = 8 Very low sample size; authors call for broader, more diverse cohorts	Proposed portable, closed loop (automatic feedback) VRET systems
[86]	Real-time anxiety-level recognition	N/A	N/A	*n* = 7 Very low sample size	Recommend integrating anxiety-level prediction into UI for therapists to monitor patient anxiety during VRET

#### Comparison of primary vs. sensitivity analyses

In the primary analysis (Low/Medium RoB ML studies), five out of eleven studies involved therapists or patients in goal setting, design, or evaluation (~45%). In the sensitivity analysis (High RoB ML studies), none (zero out of five studies) reported stakeholder involvement. This pattern underscores that higher-quality ML studies more often integrate clinical perspectives, while High RoB studies were entirely “out-of-the-loop.” Primary-analysis studies reported modest gains over clinical baselines for outcome prediction (often ~0.60–0.63 accuracy; variable AUROCs depending on multimodal inputs), with between-site generalizability frequently limited. Sensitivity-analysis studies often reported higher within-sample classification accuracies despite very small, convenience samples and minimal external validation—findings best interpreted as technical proofs-of-concept rather than clinically actionable evidence. Primary-analysis ML studies rarely articulated exposure/extinction models; a minority referenced inhibitory learning or neurocognitive correlates of extinction. Sensitivity-analysis studies largely omitted therapeutic theory, occasionally citing general emotion models (e.g., Ekman, Russell) rather than exposure/extinction frameworks.

##### Conversational AI studies

Table 5 summarizes the main findings for the Conversational AI studies.

**Table 5:** Conversational AI studies.

Authors / year	Aims	RoB
Obremski & Wienrich [77]	An AI-therapist (human-like virtual agent) controlled by OpenAI ChatGPT4 was implemented to conduct the VRET. The speech recognition and the generation of synthetic speech used Microsoft Azure in combination with Unity (3D game creation engine).	Low
Chen [78]	This study aimed to investigate the effects of technology-enhanced learning on reducing English as Foreign Language (EFL) learners’ Public Speaking Anxiety (PSA). Three groups received either lecture-based, mobile-assisted, or VR-facilitated instruction.	Low
Concannon et al. (2020) [79]	IBM Watson was linked to Unity with a script that streamed audio data from the user to the Watson speech-to-text service. Upon recognizing the question based on the speech recognition output, a virtual patient avatar would respond with a previously voice-acted recorded response.	Low
ter Heijden & Brinkman [80]	Authors created a rules-based speech recognizer that would control the dialogue in a VRET session for social anxiety. The first condition did not use any speech recognition. The rule was to use the amount of time the patient was talking to choose a response. The second condition used the speech recognizer and checked on a pre-defined finite list of keywords. The third condition checked for specific keywords appropriate for the specific point in the dialogue. The last condition was a control where a therapist manually selected the responses.	Low

#### Study aims and AI role

Overall, the conversational AI studies [77–80] examined AI applications intended to enhance VRET through virtual agents that interact with patients in VR. One study [77] designed the agent to assume the therapist’s role. To foster patient trust, the agent was gender-matched to the patient and used natural-looking gestures and facial expressions, including frequent smiling. Another study [78] compared conversational AI–driven VRET—which delivered tailored feedback based on speech and facial recognition—with a mobile app and real-life classroom lectures. Study [79] focused on enhancing the believability of VRET by using human-like agents in a simulated clinical operations exam to reduce students’ test anxiety. The earliest study [80] was a proof of concept featuring a semi-scripted conversation between virtual characters and a patient, using both manually selected and automatic speech responses, with the goal of supporting therapists and patients during VRET sessions.

#### Theories or models of extinction/exposure used

Study [77] referenced Foa and Kozak’s theory of emotional processing of fear, in which exposure corrects erroneous information embedded in a fear structure [81]. Study [79] drew on extinction-as-inhibitory-learning frameworks, citing Craske et al. [87–89] and Sotres-Bayón et al. [90]. Studies [78, 80] did not provide theoretical background on exposure or extinction.

#### How patients’/therapists’ perspectives were included

Several studies defined goals in collaboration with therapists and/or patients. Study [77] detailed an ethically guided development process for an AI-therapist (a human-like virtual agent) controlled by GPT-4 (a large multimodal transformer model developed by OpenAI) to deliver VRET. The prototype was co-developed and evaluated with a therapist. The authors followed ethical AI principles: beneficence (trust-building and bias detection), risk avoidance (risk detection), autonomy (data management), fairness (explainability, transparency, usability), and involvement (human-centeredness). A patient was also consulted during prototype testing under close therapist supervision. In study [79], the intervention was designed by an interdisciplinary team of VR learning experts, clinical evaluators, and curriculum/stress-assessment specialists. In study [80], although the main experiment involved non-patients, the authors included a case study with a patient with social phobia and a therapist to assess clinical applicability.

#### Main results and added value

In study [77], the patient testing the system reported high satisfaction with environmental realism (rating 7), involvement (9), appropriateness for therapy (8), willingness to recommend to others (9), and overall evaluation (7), suggesting high patient acceptance of AI-controlled VRET as a supplement to human-controlled VRET. However, the agent’s speech was rated lower on naturalness (3), empathy (3), and human-likeness (3).

In study [78], the authors found no statistically significant differences among the three conditions (AI + VRET, mobile app, real-life classroom lecture).

In study [80], manually selected responses did not outperform all automatic response techniques for either non-patients or patients with social phobia on presence, dialogue flow, discussion satisfaction, dialogue realism, or avatar interruption. Manual control was rated better for creating the sense that avatars were “really listening.” The therapist rated automation as beneficial because it reduced system workload and allowed greater focus on the patient.

#### Implications for clinical practice

Study [77] discussed “in-the-loop” versus “out-of-the-loop” AI for therapy. In the former, AI would trigger interventions that a therapist can define, modify, or cancel; in the latter, AI would manage the session directly without therapist involvement.

#### Study limitations

In study [77], the authors emphasized that the therapeutic relationship strongly influences outcomes and must be considered when developing AI-based VRET. If the therapist’s role is semi-automated or AI-assisted, the agent should retain memory across sessions. The authors also noted that speech-related qualities—naturalness, empathy, human-likeness—are critical for establishing a therapeutic alliance, a known predictor of psychotherapeutic success. They recommended that AI-controlled VRET incorporate multimodal inputs beyond verbal content, including physiological data, facial expressions, eye-tracking, gestures, and speech characteristics.

In study [79], which included 49 students in a clinical exam–anxiety context, the authors noted that the sample size was adequate for within-subject analyses but should be increased to strengthen between-subject inferences.

##### Knowledge-based/hybrid AI studies

Table 6 summarizes the main findings for the knowledge-based/hybrid AI studies.

**Table 6:** Knowledge-based/hybrid AI studies.

Authors / year	Aims	RoB
Heyse et al. [91]	Authors designed a concept for a semantic reasoning (rules-based AI) adaptation algorithm that would assist therapists in generating tailored VRET environments, based on the therapist’s feedback and on expectations of outcomes provided by patients at each session. The first rule of the algorithm concerned whether the patient had a hypothesis about the outcome or about the effect that an element of the VRET simulation would have on them (expected level of anxiety). The second rule calculated the value of the association change defined by the Rescorla-Wagner model, calculated from data collected through the exposure log. The Adaptor component of the system then used this information, stored in a continuously updated Knowledge Base, for personalizing the environment.	Low
Ménélas et al. [93]	In a simulation of a road tour, used with truck drivers that had been diagnosed with PTSD, cars, trucks and motorcycles appeared randomly at a chosen frequency and density and adapted their speed to the road and the road signs, respecting stop signs and traffic lights.	Low
Tartarisco et al. [92]	Authors used 139 h of valid data collected during VRET sessions designed to induce stress to extract features and rank them. For model validation, the previously collected data were manually classified as low stress, medium stress and high stress and compared to psychometric assessment results (STAY).	Medium

#### Study aims and AI role

Across these studies, goals were developed collaboratively by researchers, patients and therapists in clinical settings. Two studies [91, 92] aimed to support therapists during VRET sessions. One study [93] focused on patient agency within VRET, presenting two cases in which patients with PTSD were followed through multiple sessions. The AI component dynamically adapted the environment to patients’ responses and actions while patients verbalized their experiences and reactions during exposure. The patients were truck drivers with PTSD who operated a truck in a driving simulator with haptic feedback.

#### Theories or models of extinction/exposure used

Study [91] provided a detailed account of Pavlovian conditioning and extinction as the theoretical basis for exposure therapies. Authors applied an exposure-as–fear-reduction perspective grounded in the Rescorla–Wagner model [94].

#### How the perspectives of patients’/therapists’ were included

In study [91], three patients and two psychotherapists participated; the psychotherapists had expertise in manually designing VRET for patients. Study [93] was conducted in a therapy center with two patients diagnosed with PTSD. Study [92] involved 20 nurses; the system was designed to assist interventions for stress and anxiety in clinical workplaces and was tested in a field trial in which nurses encountered virtual scenarios mirroring workplace stressors.

#### Main results and added value

In study [93], one patient no longer required medication after the AI + VRET exposure, and another reported decreased symptoms after therapy. In study [92], the authors reported that their model classified four stress levels with an overall accuracy of approximately 83%.

#### Implications for clinical practice

The authors of study [91] noted that although their adaptive system often suggested configurations similar to those recommended by experienced therapists, it was unclear whether less experienced psychotherapists would benefit from such automation. They observed that experienced psychotherapists sometimes selected configurations that differed substantially from the automated suggestions. They argued that fully quantifying and automating the complex process of exposure therapy is unlikely; therefore, they envision their approach as a decision-support system to guide—not replace—psychotherapists. They also suggested that AI could learn from psychotherapists’ decisions over time, enabling personalization not only to patient needs but also to therapist preferences and styles.

#### Study limitations

The authors of study [91] noted that larger samples are required to validate their findings. The authors of study [92] reported that the integrated accelerometer in their monitoring setup precluded stress measurement during movement, limiting HRV-based assessment during ambulatory periods.

## Discussion

The clinical significance of this review concerns the identification of three clear patterns emerging across the literature: (i) Machine-learning (ML) systems are useful for personalizing VRET by predicting exposure schedules or classifying symptom severity, (ii) Conversational AI systems most often enhance engagement and coaching, thereby supporting both therapists and patient experience, and (iii) Knowledge-based or hybrid AI systems primarily deliver decision support and adaptive scenario configuration, combining therapist support with personalization.

Although methodological heterogeneity made it unfeasible to conduct a meta-analysis, the convergence at the use-case level (personalization, engagement, decision support) supplies clinicians, technologists and researchers with a practical taxonomy for selecting or developing AI tools.

In the remainder of this discussion, we focus on benefits, limitations, implementation barriers, practical recommendations, and future directions.

### Benefits of AI for VRET

Three key areas capture the potential benefits of AI for VRET: *personalization, engagement* and *agency*.

In terms of *personalization*, the literature on AI in mental health highlights the need to consider cultural and individual factors when determining how best to help a patient [95]. AI can be beneficial in this regard, as it can excel at integrating complex, multimodal individual measures to better personalize VRET. Several ML studies in our review addressed key considerations for this task, such as adequate sample sizes, potential biases, and outlier handling. Studies exemplifying best practices included those using theragnostic, clinical, and demographic predictors. They demonstrated the rigor needed to tackle challenges in pre- and posttreatment prediction. Notably, however, fewer than half of the ML studies involved collaboration with clinicians.

For *engagement*, it was positive to find that nearly all studies in the “conversational AI” and “knowledge-based/hybrid AI” clusters were developed in collaboration with patients and/or therapists. The potential benefits of AI for VRET were more clearly demonstrated in these human-centered studies, in which AI tools were designed to augment—rather than replace—the therapist–patient interaction. These studies contributed thoughtful discussions of ethical development, emphasizing human oversight and the use of AI to meet individual patient needs, rather than focusing solely on group-level analyses. Tangible benefits were documented, such as patients no longer needing medication and therapists expressing that the AI tools could ease their work. Some studies reported overall satisfaction scores that ranked the conversational agents as positive complements to therapy, providing validation for this AI approach.

*Agency* was a benefit that knowledge-based/hybrid AI studies pointed to. If patients and therapists feel like they can provide feedback to the system and that it continuously learns from and adapts to their needs, this might provide a greater sense of agency over the therapy. Such action/agency AI approaches align well with early descriptions of VRET (e.g., Rothbaum’s work), which conceptualized VR not as a replacement for the therapist–patient relationship but as an intermediary tool that can strengthen the therapeutic alliance [96]. These AI approaches can also contribute to increasing the acceptability of VRET more generally.

### Limitations and ethical concerns

Across studies, small, homogeneous samples and limited external validation reduce generalizability, “out-of-the-loop” automation risks undermining human oversight, and black-box models may propagate bias (few studies assessed fairness or accessibility). Where studies lacked external validation, relied on small homogeneous samples, or omitted stakeholder-defined outcomes, clinical interpretability and generalizability were limited. Also, and as mentioned earlier, the heterogeneity of included studies limits meta-analytic synthesis and complicates direct comparison of numerical performance.

Overall, ethical and equity considerations were absent from many of the studies reviewed. Explicit aims related to accessibility or inclusion were not stated in the examined studies. It is concerning that fewer than half of the ML studies in the primary analysis involved collaboration with patients or therapists to define goals, develop applications, or conduct evaluations. This issue was most salient for studies aiming at classification of symptom severity. In ML studies that did not include collaboration, researchers tended to favor solutions that made systems more independent of human intervention—what one conversational AI study termed “out-of-the-loop” AI. This conceptualization appears at odds with the therapeutic alliance, in which the therapist–patient relationship is central. It also frames AI as replacing human participation and may overlook the ongoing need for human oversight.

Several studies focused on using ML to classify patients’ responses to provide biofeedback or to adjust stimuli to participants’ baselines. However, clinical literature has highlighted challenges with such classifications. Some models function as black boxes (algorithms create internal rules that are difficult to audit due to the complexity of mathematical operations involved), making it impossible to determine which features drive classifications and, in turn, to assess potential bias in decisions such as labeling someone as having a severe disorder [97]. This can reproduce biases present in their training data. Small, homogeneous samples may result in classifications valid only for the specific groups studied and may not generalize. We address these points in more detail in the practical recommendations section below.

### Implementation barriers

Each cluster examined presents its own set of specific barriers for implementation. For ML systems, sensor burden, stewardship of multimodal streams, monitoring and external validation requirements, and clinician training to interpret probabilistic outputs and uncertainty all impede adoption. ML-based automation of VR using real‑time physiological measurements remains common, but barriers include the cost and complexity of sensors, ease of use, and ethical data governance for multimodal measures. For conversational AI, limitations in speech interpretation (accent, prosody, speech differences), empathy and human‑likeness, risks from unsupervised use, and resource demands for generative models—including constrained memory across sessions—necessitate safeguards for vulnerable populations before clinical deployment. For knowledge‑based/hybrid decision support, addressing the need for rule transparency and understanding high maintenance burdens (periodic updates and verification of knowledge bases) is essential. Also, semantic rules must align with therapist styles to avoid over‑automation and preserve clinical judgment. Beyond cluster‑specific issues, privacy and security concerns—particularly if integrating born‑digital records—must be carefully assessed before any large‑scale use in therapy or diagnosis [97].

### Theoretical grounding of AI‑enhanced VRET - an underused aspect

Across applied studies, contemporary exposure/extinction frameworks were underutilized. Exposure/extinction models (inhibitory learning, emotional processing) are rarely embedded in AI logic. Incorporating expectancy violation, variability, and context shifts could boost efficacy. Inhibitory learning models (Craske et al. [87]) emphasize expectancy violation, variability, and spacing; contextual renewal and generalization research highlights the value of cue variability and retrieval cues; emotional processing theory (Foa & Kozak [81]) underscores updating fear structures via corrective information. Embedding these principles in AI logic could improve clinical relevance. For example, implementing expectancy violation through AI could help target patient‑specific predictions (self‑report + physiology) to engineer safe mismatches (e.g., variable schedules, unpredictable but bounded intensity changes). Algorithmically optimizing stimulus variability and inter‑trial intervals to prevent overfitting to narrow cues and support generalization could enhance the variability aspect of the exposure schedule. Incorporating context shifts and retrieval cues within and across sessions through AI automation could reduce relapse via renewal. Also, AI could help identify which theoretical evidence would best match a given symptomatology, to then provide suggestions for the exposure hierarchies, schedules and other aspects of the VRET treatment. Keeping therapists in the loop for overrides to the AI aspects of the VRET system would maintain an ethical approach to AI for VRET - human supervision would be engineered into the system to ensure that arousal levels do not cross safety thresholds.

### Practical recommendations

Practical areas that could be prioritized include (i) following emerging AI-ethics guidance (EU AI Act, APA statements), (ii) providing model explainability, calibration, and subgroup error analyses, (iii) using co-design workshops involving patients from different backgrounds, clinicians, regulators, members of professional psychology associations working on developing VRET + AI automation guidelines, AI/VR engineers, designers, data security/privacy experts, clinic technicians, and researchers. This type of transdisciplinary collaboration can help create solid frameworks for cross-site validation. Furthermore, such exchanges can contribute to improve clinician and patient AI-literacy, and conversely, improve engineers’ and designers’ understanding of the needs of patients and therapists.

For point (i), future studies should consider guidance such as the European Commission’s Living Guidelines on the Responsible Use of Generative AI [98] and the European Union’s AI Act [99]. Several reviews of VRET [100, 101] have emphasized the need for closer collaboration among technology developers, researchers, and stakeholders to address outcome needs and acceptability across diverse participant groups, while also highlighting that practitioner training is insufficiently available. The American Psychological Association has also recently released specific ethical guidance for AI in mental healthcare [102, 103] where they recommend that healthcare professionals explain to patients the fundamental differences between interacting with a conversational agent (generative AI chatbot). Patients, especially children, adolescent and vulnerable populations, should not be left alone to use these technologies as replacement of professional therapists, due to the risks of unhealthy attachment or other possible negative effects.

For point (ii), clinically feasible interpretability and governance of AI for VRET could be ensured through model‑agnostic explainability (e.g., SHAP values [104], permutation feature importance) to surface drivers of predictions. This could be complemented by the presentation of clinician‑readable summaries of top features and uncertainty in the models. Calibration (reliability curves) and decision thresholds should be reported and should align with clinical risk tolerances. Subgroup error analyses (by age, gender identity/expression, ethnicity, comorbidity) should be performed to detect inequities, and fairness metrics could be included where feasible. In general, simpler or hybrid interpretable models should be used when performance is comparable; for deep models, post‑hoc explanations plus guardrails (confidence gating, override rules) should be provided. In addition, model cards and data sheets summarizing provenance, intended use, and known limitations should be provided, and audit trails for clinical decisions informed by AI should be maintained. Where possible, external (cross‑site) and prospective validation should be performed, and deployment based solely on single‑site convenience samples should be avoided.

For point (iii) on transdisciplinary collaboration, effective AI‑enabled VRET requires co‑creation among clinical psychologists and psychiatrists, software engineering and data science/ML professionals, human‑computer interaction/UX designers, AI ethics and law regulators, privacy and security experts, health equity specialists, researchers working in the fields of VRET and fear and anxiety disorders, and patient advocacy groups. One of the biggest challenges in transdisciplinary collaboration is the translation of concepts and approaches between disciplines. Experts in AI and experts in the psychology of fear and anxiety disorders need to formulate a common language (at a more general level, this point has been raised by [105] in the Marburg Declaration as an ongoing need in mental healthcare). This also involves feedback from patients so that the applications developed are truly addressing their needs. Addressing bias in AI requires intentionally including a broad spectrum of users and ensuring diversity with respect to accessibility needs, gender identity and expression, ethnicity, age, culture, and socioeconomic background. The process should be a multi-directional dialogue that invites all these communities to contribute. The emergence of a common vocabulary and set of goals could be achieved through several interconnected pathways. One pathway could include co‑design workshops and participatory action research with patients and therapists, as successfully demonstrated by several studies in this review [77, 80, 91–93]. A second pathway could be the promotion of joint VRET + AI ethics and safety review boards at regional or national levels, which could be initiated by the communities of interested stakeholders (for example, professional psychology associations and representatives for mental health patient groups). A third pathway could be the development of training curricula for mental health clinicians focusing on AI literacy and risk/benefit interpretation. We believe this task must be prioritized by higher education organizations. Finally, a fourth pathway could involve broader community involvement through consultations, community advisory boards and information sessions involving both patients and their next of kin. Such community-based fora could help AI technology developers, researchers and clinicians identify equity and accessibility concerns. If some of these pathways are initiated, we believe a transdisciplinary field will emerge, which could help bridge the clinical gaps and translational challenges addressed in this review.

### Future directions

Four areas could be prioritized as future directions for research: (i) the creation of large, multi-site datasets for robust ML models, (ii) the development of theory-driven AI that automates expectancy violation and stimulus variability, (iii) conducting head-to-head trials comparing AI-enabled VRET with traditional VRET and (iv) supporting transdisciplinary collaboration through research that involves clinicians, engineers, ethicists, and patient advocates.

Point (i) was suggested by some of the ML studies, which called for the creation of large multi-site studies to investigate how multiple features, including multimodal measures, could be combined to create larger, more robust datasets for training.

Point (ii) concerns increasing the sophistication of VRET stimuli and contexts, aligned with contemporary exposure/extinction theory. While several studies discussed existing theories, in general the studies relied on older conceptualizations for developing their AI concepts, even when citing some of the more up-to-date theories. There seemed to be restricted integration between the applied AI and VRET studies and theoretical and experimental research on exposure/extinction. There was no clear pattern of correspondence between the theories cited and the dates of publication, and studies using emotion processing theories were interspersed with studies using inhibitory learning theories. Further sophistication of conceptualizations for AI-enhanced VRET could connect more closely to the latest theoretical developments. Applying theory-driven strategies like positive counterconditioning and incorporating a broader range of stimuli and contexts may strengthen generalization effects, thereby improving therapeutic outcomes.

Point (iii) should be a priority for future studies. Comparing AI-enabled VRET with other VRET modalities is essential to determine whether AI-enabled VRET is the appropriate intervention for a given case, yet none of the studies in our review addressed this directly.

Lastly, in point (iv), we come back to the need for more transdisciplinary collaboration. Future research could include pathways for software engineers, clinical users, experienced VRET therapists, and researchers to collaborate in AI-enhanced VRET. Part of this work could focus on the AI training needs of therapists. This could also be beneficial for increasing the use of ET in general, which despite strong support, and as stated in the introduction, is still low. We hope this review can contribute to this task by serving as a practical guide for transdisciplinary teams—clinicians, software engineers/ML scientists, HCI/UX designers, AI ethics and legal experts, privacy/security leads, health equity specialists, and patient advocates—to co‑design AI‑enabled VRET that is theory‑informed, interpretable, safe, and equitable.

## Supplementary information

Table S2 - technical features of included studies

Table S1- recent reviews and meta-analyses of VRET

RoB Assesment

List of AI literature, Supplemmentary Materials AI VRET
