# Supplementary material for: Artificial intelligence (AI) for virtual reality exposure therapy (VRET): A systematic review
Source: Transl Psychiatry. 2026 Mar 26;16:208. doi: 10.1038/s41398-026-03936-4 (PMC13039931; doi:10.1038/s41398-026-03936-4)
Supplement: Supplementary file 1 — Table S2 - technical features of included studies [file 41398_2026_3936_MOESM1_ESM.pdf]

Table S2 – Technical characteristics of included studies

ML Studies

Authors / year	Type of AI
Jung et al. (2025) [64]	GNB, k-NN, LRR, SVC, RF, and SGB.
Chavanne et al. (2023) [65]	RF.
Cheng et al. (2023) [66]	k-NN, extra trees, RF, SVM and logistic regression.
de With et al. (2022) [67]	LDA and SVM
Leehr et al. (2021) [68]	RF and out-of-bag (oob) accuracy tests.
Park et al. (2025) [69]	RF, XGBoost, l LightGBM, and CatBoost.
Apicella et al. (2024) [70]	DA, data fusion and participant clustering strategies for RF, SVM, k-NN and DNN.
Apicella et al. [71]	k-NN, RF, GNB, and SVM.
Goel et al. (2023) [72]	SVM, SVR, and MLP.
Chun et al. (2022) [73]	Logistic regression, RF, and GNB.
Handouzi et al. (2014) [74]	SVM.
Mevlevioğlu et al. (2024) [75]	ANN, SVM, k-NN, CNN.
Rahman et al. (2023). [76]	GNB, QDA, SVM, MLP, ADB, k-NN, DT and RF.
Petrescu et al. (2020) [77]	Machine Learning algorithms and CNNs.
Bălan et al. (2020) [78]	k-NN, SVM with linear kernel, RF, LDA, and 4 DNN models.
Šalkevicius et al. (2019) [79]	SVM.

Abbreviations: logistic ridge regression (LRR), support vector classifier (SVC), stochastic gradient boosting (SGB), artificial neural network (ANN), support vector machine (SVM), k-nearest neighbors (k-NN), convolutional neural network (CNN), Gaussian naive Bayes (GNB), quadratic discriminant analysis (QDA), linear discriminant analysis (LDA), multilayer perceptron (MLP), AdaBoost (ADB), decision tree (DT), random forest (RF), instance-based learning (IBL), self-organizing map (SOM), Gaussian process classifier (GPC), extremely randomized trees (ExtraTrees), extreme gradient

boosting (XGBoost), Light Gradient Boosting Machine (LightGBM), Categorical Boosting (CatBoost), support vector regression (SVR), domain adaptation (DA), deep neural networks (DNN).

Conversational AI

Authors / year	Type of AI
Obremski & Wienrich (2024) [82]	Conversational AI using Generative AI with LLMs - OpenAI's ChatGPT4
Chen (2022) [83]	The researchers used VirtualSpeech, an app that creates realistic scenarios and provides constant feedback through speech analysis.
Concannon et al. (2020) [84]	Conversational AI using IBM Watson
ter Heijden & Brinkman (2011) [85]	Scripted Speech Recognition. While this study is not strictly using AI, we include it as an example of the early attempts to create Conversational AI for VRET.

Abbreviations: Large Language Models (LLM)

Knowledge-based/hybrid AI

Authors / year	Type of AI
Heyse et al. (2022) [91]	Semantic Reasoning (Rules-based AI).
Ménélas et al. (2018) [92]	Artificial Intelligence in VRET simulation control.
Tartarisco et al. (2015) [93]	ReliefF algorithm and Davies-Bouldin (DB) cluster evaluation index. Features were inputs for a neuro-fuzzy model (SOM + rule-based fuzzy model). Thereafter compared with IBL, GNB, MLP, J48, RF, single SOM and SVM.

Abbreviations: artificial neural network (ANN), support vector machine (SVM), multilayer perceptron (MLP), random forest (RF), instance-based learning (IBL), self-organizing map (SOM), Gaussian naive Bayes (GNB), J48 (Java implementation of the C4.5 algorithm). A list of recommended in-depth references for these terms is provided in the Supplementary Materials.
