# Supplementary material for: Artificial intelligence (AI) for virtual reality exposure therapy (VRET): A systematic review
Source: Transl Psychiatry. 2026 Mar 26;16:208. doi: 10.1038/s41398-026-03936-4 (PMC13039931; doi:10.1038/s41398-026-03936-4)
Supplement: Supplementary file 2 — Table S1- recent reviews and meta-analyses of VRET [file 41398_2026_3936_MOESM2_ESM.pdf]

Supplementary materials 1

Recent reviews and meta-analyses on VRET

Several reviews and meta-analyses have assessed the benefits of VRET [21–33], finding it effective in producing desired affective outcomes for anxiety and several specific phobias when compared with *in vivo* exposure and control conditions, and identifying it as a useful approach for studying questions such as the combined effects of pharmacological compounds and behavioral manipulations. Reviews also highlight unresolved questions about how spatial and social presence contribute to treatment efficacy, and about the effectiveness of VRET for patients with limited imagery ability [40]. In this context, effectiveness refers to the ability of VRET to deliver the right type of exposure and achieve the right type of symptom relief in patients; efficacy refers to benefits under controlled conditions and the capacity of VRET to deliver a complete exposure protocol; and efficiency refers to practical considerations such as convenience, cost, and time.

Table S1 presents an overview of relevant reviews and meta-analyses on VRET.

Table S1. Overview of systematic reviews, literature reviews and meta-analyses on VRET

Authors	Population	Comparison condition	Outcomes
Valmaggia et al. [34]	Systematic review of 24 RCT studies	Effectiveness of VRET vs CBT vs Treatment As Usual vs Waiting List	Found VRET to be more effective than treatment as usual or waiting list control, and VRET had similar effectiveness when compared to conventional Cognitive Behavioral Therapy (CBT) and *in vivo* exposure
Cardoş et al. [35]	Meta-analysis of 11 RCT studies	Efficiency of VRET vs Control vs Classical Evidence-Based Interventions	Significant overall efficiency of VRET in flight anxiety at post-test and follow-up when compared to control and classical evidence-based interventions and found similar efficacy between VRET and other exposure-based interventions.

		vs Other Exposure-based Interventions	
Wechsler, T.F., F. Kumpers, and A. Muhlberger [36]	Nine studies (n = 371) included, four on Specific Phobia, three on Social Phobia, and two on Agoraphobia.	Effect size of VRET vs *In Vivo* Exposure	A review looking specifically at agoraphobia, specific phobia and social phobia, where authors reported that both VRET and *in vivo* exposure showed large, significant effect sizes, with a small but non-significant difference favouring *in vivo* over VRET
Deng et al. [37]	18 studies on PTSD: 13 RCTs (n = 654) and 5 single-group trials (n = 60)	Effect of VRET in PTSD	Found that VRET had a moderate effect on PTSD symptoms.
Kothgassner and Felnhofer [38]	4 trials (n = 100) of children and adolescents ages 8 and 16 years	Clinical improvement of symptoms after VRET	Found evidence of clinical improvements in symptoms of anxiety disorders in children and adolescents after VRET, but found also that there was a lack of controlled trials for younger cohorts.
Emmelkamp et al. [39]	Narrative review of RCT studies, meta-analyses and other types of studies on VRET for SAD	VRET on SAD	Conducted a literature review on VRET for Social Anxiety Disorder (SAD) and found there was a lack of studies investigating the effect of VRET as a stand-alone treatment for SAD, and that most RCTs investigating the efficacy of VRET for SAD combined the treatment with other cognitive interventions.
Chard and van Zalk [40]	12 studies with clinical and subclinical SAD populations	VRET on SAD	In terms of the effectiveness of VRET for SAD, authors pointed out the need to further investigate therapist guided VRET vs. automated VRET.
Knaust, T., et al. [41]	Scoping review of eighteen	VRET on PTSD	Pointed out that the efficacy of spatial or social presence and the efficacy of VRET for patients with imagination difficulties still needed to be addressed.

	studies (RCT, case studies and pilot study) of VRET for PTSD		

References (follow numbering of main article)

21. Parsons TD, Rizzo AA. Affective outcomes of virtual reality exposure therapy for anxiety and specific phobias: a meta-analysis. J Behav Ther Exp Psychiatry 2008; 39: 250–261.
22. Powers MB, Emmelkamp PM. Virtual reality exposure therapy for anxiety disorders: a meta-analysis. J Anxiety Disord 2008; 22: 561–569.
23. Botella C, et al. Recent progress in virtual reality exposure therapy for phobias: a systematic review. Curr Psychiatry Rep 2017; 19: 42.
24. Carl E, et al. Virtual reality exposure therapy for anxiety and related disorders: a meta-analysis of randomized controlled trials. J Anxiety Disord 2019; 61: 27–36.
25. Horigome T, et al. Virtual reality exposure therapy for social anxiety disorder: a systematic review and meta-analysis. Psychol Med 2020; 50: 2487–2497.
26. Ioannou A, et al. Virtual reality and symptoms management of anxiety, depression, fatigue, and pain: a systematic review. SAGE Open Nurs 2020; 6: 2377960820936163.
27. Freitas JRS, et al. Virtual reality exposure treatment in phobias: a systematic review. Psychiatr Q 2021; 92: 1685–1710.

28. Reeves R, et al. A meta-analysis of the efficacy of virtual reality and in vivo exposure therapy as psychological interventions for public speaking anxiety. Behav Modif 2022; 46: 937–965.
29. Krzystanek M, et al. Tips for effective implementation of virtual reality exposure therapy in phobias—a systematic review. Front Psychiatry 2021; 12: 737351.
30. Eshuis LV, et al. Efficacy of immersive PTSD treatments: a systematic review of virtual and augmented reality exposure therapy and a meta-analysis of virtual reality exposure therapy. J Psychiatr Res 2021; 143: 516–527.
31. Kothgassner OD, et al. Virtual reality exposure therapy for posttraumatic stress disorder (PTSD): a meta-analysis. Eur J Psychotraumatol 2019; 10: 1654782.
32. Morina N, et al. Can virtual reality exposure therapy gains be generalized to real-life? A meta-analysis of studies applying behavioral assessments. Behav Res Ther 2015; 74: 18–24.
33. Valmaggia LR, et al. Virtual reality in the psychological treatment for mental health problems: a systematic review of recent evidence. Psychiatry Res 2016; 236: 189–195.
34. Cardoş RAI, David OA, David DO. Virtual reality exposure therapy in flight anxiety: a quantitative meta-analysis. Comput Human Behav 2017; 72: 371–380.
35. Wechsler TF, Kumpers F, Muhlberger A. Inferiority or even superiority of virtual reality exposure therapy in phobias? A systematic review and quantitative meta-analysis on randomized controlled trials specifically comparing the efficacy of virtual reality exposure to gold standard in vivo exposure in agoraphobia, specific phobia, and social phobia. Front Psychol 2019; 10: 1758.

36. Deng W, et al. The efficacy of virtual reality exposure therapy for PTSD symptoms: a systematic review and meta-analysis. J Affect Disord 2019; 257: 698–709.
37. Kothgassner OD, Felnhofer A. Lack of research on efficacy of virtual reality exposure therapy (VRET) for anxiety disorders in children and adolescents: a systematic review. Neuropsychiatr 2021; 35: 68–75.
38. Emmelkamp PMG, Meyerbroker K, Morina N. Virtual reality therapy in social anxiety disorder. Curr Psychiatry Rep 2020; 22: 32.
39. Chard I, van Zalk N. Virtual reality exposure therapy for treating social anxiety: a scoping review of treatment designs and adaptation to stuttering. Front Digit Health 2022; 4: 842460.
40. Knaust T, et al. Virtual trauma interventions for the treatment of post-traumatic stress disorders: a scoping review. Front Psychol 2020; 11: 562506.
