# Supplementary material for: Artificial intelligence (AI) for virtual reality exposure therapy (VRET): A systematic review
Source: Transl Psychiatry. 2026 Mar 26;16:208. doi: 10.1038/s41398-026-03936-4 (PMC13039931; doi:10.1038/s41398-026-03936-4)
Supplement: Supplementary file 3 — RoB Assesment [file 41398_2026_3936_MOESM3_ESM.pdf]

Artificial Intelligence (AI) for Virtual Reality Exposure Therapy (VRET): A Systematic Review

Authors: Kamilla Bergsnev, Ana Luisa Sánchez Laws

RISK OF BIAS / QUALITY ASSESSMENTS

JBI Critical Appraisal Checklist for case reports

Moola S, Munn Z, Tufanaru C, Aromataris E, Sears K, Sfetcu R, Currie M, Qureshi R, Mattis P, Lisy K, Mu P-F. Chapter 7: Systematic reviews of etiology and risk. In: Aromataris E, Munn Z (Editors). JBI Manual for Evidence Synthesis. JBI, 2020. Available from https://synthesismanual.jbi.global

Authors	Were patient's demographic characteristics clearly described?	Was the patient's history clearly described and presented as a timeline?	Was the current clinical condition of the patient on presentation clearly described?	Were diagnostic tests or assessment methods and the results clearly described?	Was the intervention(s) or treatment procedure(s) clearly described?	Was the post-intervention clinical condition clearly described?	Were adverse events (harms) or unanticipated events identified and described?	Does the case report provide takeaway lessons?	**TOTAL**	**Subjective evaluation**
Obremski & Wienrich	1	1	1	1	1	1	1	1	8	low RoB
Heyse et al.	1	1	1	1	1	1	1	1	8	low RoB
Menelas et al.	1	1	1	1	1	1	1	1	8	low RoB

JBI Critical Appraisal Checklist for cohort studies

Moola S, Munn Z, Tufanaru C, Aromataris E, Sears K, Sfetcu R, Currie M, Qureshi R, Mattis P, Lisy K, Mu P-F. Chapter 7: Systematic reviews of etiology and risk . In: Aromataris E, Munn Z (Editors). JBI Manual for Evidence Synthesis. JBI, 2020. Available from https://synthesismanual.jbi.global

Authors	Were the two groups similar and recruited from the same population?	Were the exposures measured similarly to assign people to both exposed and unexposed groups?	Was the exposure measured in a valid and reliable way?	Were confounding factors identified?	Were strategies to deal with confounding factors stated?	Were the groups/participants free of the outcome at the start of the study (or at the moment of exposure)?	Were the outcomes measured in a valid and reliable way?	Was the follow up time reported and sufficient to be long enough for outcomes to occur?	Was follow up complete, and if not, were the reasons to loss to follow up described and explored?	Were strategies to address incomplete follow up utilized?	Was appropriate statistical analysis used?	**TOTAL**	**Subjective evaluation**
Concannon et al.	1	1	1	1	1	1	1	1	1	1	1	11	low RoB

JBI Critical Appraisal Checklist for Qualitative Research

Lockwood C, Munn Z, Porritt K. Qualitative research synthesis: methodological guidance for systematic reviewers utilizing meta-aggregation. Int J Evid Based Healthc. 2015;13(3):179–187

Authors	Is there congruity between the stated philosophical perspective and the research methodology?	Is there congruity between the research methodology and the research question or objectives?	Is there congruity between the research methodology and the methods used to collect data?	Is there congruity between the research methodology and the representation and analysis of data?	Is there congruity between the research methodology and the interpretation of results?	Is there a statement locating the researcher culturally or theoretically?	Is the influence of the researcher on the research, and vice-versa, addressed?	Are participants, and their voices, adequately represented?	Is the research ethical according to current criteria or, for recent studies, and is there evidence of ethical	Do the conclusions drawn in the research report flow from the analysis, or interpretation, of the data?	**TOTAL**	**Subjective evaluation**

									approval by an appropriate body?			
ter Heijden & Brinkman	0	1	1	1	1	0	0	1	1	1	7	low RoB

JBI CRITICAL APPRAISAL TOOL FOR assessment of risk of bias for randomized controlled trials (2023)

Barker TH, Stone JC, Sears K, Klugar M, Tufanaru C, Leonardi-Bee J, Aromataris E, Munn Z. The revised JBI critical appraisal tool for the assessment of risk of bias for randomized controlled trials. JBI Evidence Synthesis. 2023;21(3):494-506

Authors	Was true randomization used for assignment of participants to treatment groups?	Was allocation to treatment groups concealed?	Were treatment groups similar at the baseline?	Were participants blind to treatment assignment?	Were those delivering the treatment blind to treatment assignment?	Were treatment groups treated identically other than the intervention of interest?	Were outcome assessors blind to treatment assignment?	Were outcomes measured in the same way for treatment groups?	Were outcomes measured in a reliable way	Was follow up complete and if not, were differences between groups in terms of their follow up adequately described and analysed?	Were participants analysed in the groups to which they were randomized?	Was appropriate statistical analysis used?	Was the trial design appropriate and any deviations from the standard RCT design (individual randomization, parallel groups) accounted for in the conduct and analysis of the trial?	**TOTAL**	**Subjective evaluation of risk of bias (RoB)**
Leehr et al.	1	1	1	1	1	1	1	1	1	1	1	1	1	13	low RoB

Chavanne et al.	1	1	1	1	1	1	1	1	1	1	1	1	1	13	low RoB
Tartarisco et al.	1	0	1	0	0	1	0	1	1	1	1	1	1	9	medium RoB
Chun et al.	1	1	1	1	0	1	0	1	0	0	1	1	1	9	medium RoB
Handouzi et al.	0	0	1	1	0	1	0	1	1	0	1	1	1	8	medium RoB
Balan et al.	0	0	1	1	0	1	0	1	0	0	1	1	0	6	high RoB
Mevlevioglu et al.	0	0	1	1	0	1	0	1	0	0	1	1	0	6	high RoB
Petrescu et al.	0	0	1	1	0	1	0	1	0	0	1	1	0	6	high RoB
Salkevicius et al.	0	0	1	1	0	1	0	1	0	0	1	1	0	6	high RoB
Park et al	0	0	1	1	0	1	0	1	1	0	1	1	0	7	medium RoB
Chen et al	1	1	1	1	0	1	0	1	1	1	1	1	1	11	low RoB
Jung et al	0	0	1	1	0	1	0	1	1	0	1	1	0	7	medium RoB
Goel et al	0	0	1	1	0	1	0	1	1	0	1	1	0	7	medium RoB

JBI checklist for quasi-experimental studies 2023

Barker TH, Habibi N, Aromataris E, Stone JC, Leonardi-Bee J, Sears K, et al. The revised JBI critical appraisal tool for the assessment of risk of bias quasi-experimental studies. JBI Evid Synth. 2024;22(3):378-88.

	Is it clear in the study what is the	Was there a control group?	Were participants included in any	Were the participants included in any compariso	Were there multiple measurements of the outcome, both pre and	Were the outcomes of participants included in any	Were outcomes measured in a	Was follow-up complete and if not,	Was appropriate statistical	**TOTAL**	**Subjective evaluation of risk**

	“cause” and what is the “effect” (i.e. there is no confusion about which variable comes first)?		comparisons similar?	ns receiving similar treatment/ care, other than the exposure or intervention of interest?	post the intervention/exposure?	comparisons measured in the same way?	reliable way?	were differences between groups in terms of their follow-up adequately described and analyzed?	analysis used?		**of bias (RoB)**
Rahman et al.	1	0	0	0	1	1	0	0	1	4	high RoB
Cheng et al.	1	0	1	1	1	1	0	0	1	6	medium RoB
de With et al.	1	1	1	1	1	1	1	1	1	9	low RoB
Apicella et al. 2023	1	0	1	1	0	1	1	0	1	6	medium RoB
Apicella et al. 2024	1	0	1	1	0	1	1	0	1	6	medium RoB
