# Supplementary material for: Artificial intelligence (AI) for virtual reality exposure therapy (VRET): A systematic review
Source: Transl Psychiatry. 2026 Mar 26;16:208. doi: 10.1038/s41398-026-03936-4 (PMC13039931; doi:10.1038/s41398-026-03936-4)
Supplement: Supplementary file 4 — List of AI literature, Supplemmentary Materials AI VRET [file 41398_2026_3936_MOESM4_ESM.docx]

**List of recommended books for AI/ML**

The following books should provide interested readers with a solid foundation to understand basic concepts in AI and ML.

Deisenroth, M. P., Deisenroth, M. P., Faisal, A. A., Ong, C. S., Deisenroth, Marc Peter, Faisal, A. Aldo, & Ong, Cheng Soon. (2020). *Mathematics for machine learning*. Cambridge University Press.

Goodfellow, I., Bengio, Y. , Courville,A., & Bach, F. (2016). *Deep Learning*. The MIT Press.

James, G., Witten, D., Hastie, T. J., Tibshirani, R. J., & Taylor, J. E. (2023). *An introduction to statistical learning : with applications in Python* (1st ed.). Springer. https://doi.org/10.1007/978-3-031-38747-0

Russell, S., & Norvig, P. (2021). *Artificial intelligence : a modern approach* (4th edition, Global edition.). Pearson.
